# The dissociations of visual processing of “hole” and “no‐hole” stimuli: An functional magnetic resonance imaging study

**DOI:** 10.1002/brb3.979

**Published:** 2018-04-18

**Authors:** Qianli Meng, Yan Huang, Ding Cui, Lixia He, Lin Chen, Yuanye Ma, Xudong Zhao

**Affiliations:** ^1^ State Key Laboratory of Brain and Cognitive Science Institute of Biophysics Chinese Academy of Sciences Beijing China; ^2^ CAS Center for Excellence in Brain Science and Intelligence Technology Beijing China; ^3^ University of Chinese Academy of Sciences Beijing China; ^4^ The Brain Cognition & Brain Disease Institute for Collaboration Research of SIAT at CAS and the McGovern Institute at MIT Shenzhen Institutes of Advanced Technology Chinese Academy of Sciences University Town of Shenzhen Shenzhen China; ^5^ Paralign Inc. San Francisco California; ^6^ Yunnan Key Laboratory of Primate Biomedical Research Kunming University of Science and Technology Kunming China

**Keywords:** backward masking, cortical pathway, functional magnetic resonance imaging, hole feature, subcortical pathway

## Abstract

**Introduction:**

“Where to begin” is a fundamental question of vision. A “Global‐first” topological approach proposed that the first step in object representation was to extract topological properties, especially whether the object had a hole or not. Numerous psychophysical studies found that the hole (closure) could be rapidly recognized by visual system as a primitive property. However, neuroimaging studies showed that the temporal lobe (IT), which lied at a late stage of ventral pathway, was involved as a dedicated region. It appeared paradoxical that IT served as a key region for processing the early component of visual information. Did there exist a distinct fast route to transit hole information to IT? We hypothesized that a fast noncortical pathway might participate in processing holes.

**Methods:**

To address this issue, a backward masking paradigm combined with functional magnetic resonance imaging (fMRI) was applied to measure neural responses to hole and no‐hole stimuli in anatomically defined cortical and subcortical regions of interest (ROIs) under different visual awareness levels by modulating masking delays.

**Results:**

For no‐hole stimuli, the neural activation of cortical sites was greatly attenuated when the no‐hole perception was impaired by strong masking, whereas an enhanced neural response to hole stimuli in non‐cortical sites was obtained when the stimulus was rendered more invisible.

**Conclusions:**

The results suggested that whereas the cortical route was required to drive a perceptual response for no‐hole stimuli, a subcortical route might be involved in coding the hole feature, resulting in a rapid hole perception in primitive vision.

## 1 INTRODUCTION

“What are the primitives of visual perception” is a fundamental question of vision. The viewpoint of Gestalt psychology claimed that perceptual processing was from global to local. However, Gestalt evidence had often been criticized for being mainly phenomenological and relying mainly on conscious experience (Pomerantz, 1981, 2003). A proper formal analysis of visual perception that went beyond intuitive approaches was needed to provide a theoretical basis for precisely describing or defining the core concepts related to visual perception, for example, “global” versus “local”. Based on a fairly large set of data on visual perception, a theory of “Global‐first” topology had been put forward. According to the theory, topological properties, as global properties, were the primitives of visual perception. The first step in object representation is to extract topological properties, particularly, to determine whether the object has a hole (closure) or not (Chen, 1982, 2005; Chen, Zhang, & Srinivasan, 2003; Wang, Zhou, Zhuo, & Chen, 2007; Zhou, Luo, Zhou, Zhuo, & Chen, 2010; Zhuo et al., 2003). It should be mentioned that the concept of a “hole” in this study meant a two‐dimensional concept, which did not require any extended surface, or figure‐ground structure. In this sense, the concept of a “hole” in the present was same as the concept of “closure” in the Gestalt theory. Thus, our definition of the “hole” was fundamentally different from how it has been defined in previous studies on “hole” perception, in which the “hole” has been defined as a background region that is surrounded by a foreground figure.

A large number of psychophysical studies found that early visual computations were sensitive to the hole (closure) feature, which could be rapidly recognized by the visual system as a whole simple or primitive property and maintained this advantage over other geometrical information during subsequent conscious visual perception (Bertamini, 2006; Bertamini & Lawson, 2006; Donnelly, Humphreys, & Riddoch, 1991; Elder & Zucker, 1993, 1994; Horowitz & Kuzmova, 2011; Kimchi, 1994; Kimchi & Bloch, 1998; Mark & Branka, 2011; Nelson & Palmer, 2001; Pomerantz, Sager, & Stoever, 1977; Spehar, 2002; Treisman & Patterson, 1984). For instance, Elder and Zucker found that in a task of two‐dimensional shape recognition, participants' reaction times were shorter for closed stimuli than for open ones (Elder & Zucker, 1993, 1994).

Further neuroimaging studies investigating the neural substrate of hole showed that the temporal lobe (IT), which lies in the late destination of the ventral visual pathway, is a key region for coding the hole feature (Wang et al., 2007; Zhuo et al. 2003). Moreover, a single‐unit recording study on monkeys found that neurons in the inferior temporal (IT) cortex responded selectively to “holes” with a short latency (<100 ms) (Komatsu & Ideura, 1993). However, in the perspective of classic visual pathways, the anterior temporal lobe (ATL) lied at the late stage of the ventral pathway, and thus, it appeared paradoxical that the ATL would be the key region for processing early components of visual information. Did there exist a direct and fast route transmitting the information of a hole to the IT during early vision? Some available research suggested that perceiving hole features might be a fundamental and conservative function of vision, independent of the mature cortical visual system. For example, results from a study on infants suggested that newborn babies had the ability to recognize hole features 2–3 days after birth, despite their functionally immature visual cortexes (Turati, Simion, & Zanon, 2003). Among rodent species, mice were generally not considered to be “visual animals” because of retinal degeneration and lack of infoldings in the cortex, but mice could successfully extract hole features during visual perception (Zhou et al., 2010). In addition, bees, which are ancient animals that lack a cortical system and have fewer than 0.01% of the number of neurons that the human brain contains, could also discriminate hole features (Chen, Zhang & Srinivasan, 2003).

Inspired by these findings, it was suggested that a noncortical system might be involved in the processing of holes. It is well established that, in addition to the classical cortical route, a subcortical route is also responsible for visual processing (Morris, Ohman, & Dolan, 1998, 1999; Pasley, Mayes, & Schultz, 2004; Tamietto & de Gelder, 2010). The subcortical route contains only a small number of fibers originating from the retina, and these fibers take a secondary route to the superior colliculus (SC) and the pulvinar. Functionally, the classical cortical route is known to be a slow and detailed route used to process visual stimuli for conscious identification. The visual subcortical route, however, is a rapid pathway for processing coarse and early visual component or some unconscious information, especially with some special meaning (e.g., face or emotion). It was hypothesized that the subcortical route, instead of the traditional cortical pathway, might be involved in the coding of hole features.

To test the hypothesis, we modulated the stimulus awareness levels through the backward masking paradigm and measured the neural activity in anatomically defined regions of interest (ROIs) using functional magnetic resonance imaging (fMRI). The ROIs were the early visual cortex and lateral geniculate nucleus (LGN), the SC, and the pulvinar. The stimulus onset asynchrony (SOA) between the target and the backward noise was manipulated at two levels (34 and 200 ms) (Enns & Di Lollo, 2000; Lamme, Zipser, & Spekreijse, 2002). The behavioral results indicated that in the short SOA (34 ms) condition, the visibility of hole and no‐hole stimuli was both greatly impaired by a strong masking effect. However, under these conditions, participants' performance for hole discrimination was significantly better than for no‐hole stimuli. Further ROI analysis revealed that whereas the neural response to no‐hole stimuli in LGN and early visual cortex was decreased when it was rendered more invisible, an increased neural signal to hole stimuli was observed in SC and pulvinar. Moreover, the neural activities to hole stimuli in the SC, pulvinar, and early visual cortex were greater than no‐hole stimuli under strong masking conditions. These findings suggested that whereas a cortical route, via LGN and early visual cortex, was required for no‐hole stimuli to “drive” a perceptual response, the subcortical pathway through SC and pulvinar might be involved in coding the hole feature, especially under low awareness conditions.

## 2 METHOD

### 2.1 Participants

Nineteen volunteers (eight males and 11 females, aged at 21–29 years old) participated in this study and received money compensation for the time they spent. All had normal or corrected to normal vision and were right‐handed. Written informed consent was obtained from all participants in accordance with requirements of the Institutional Review Board of the Beijing MRI Center for Brain Research and was treated in accordance with the Declaration of Helsinki.

### 2.2 Stimuli and procedure

**Figure 1:**
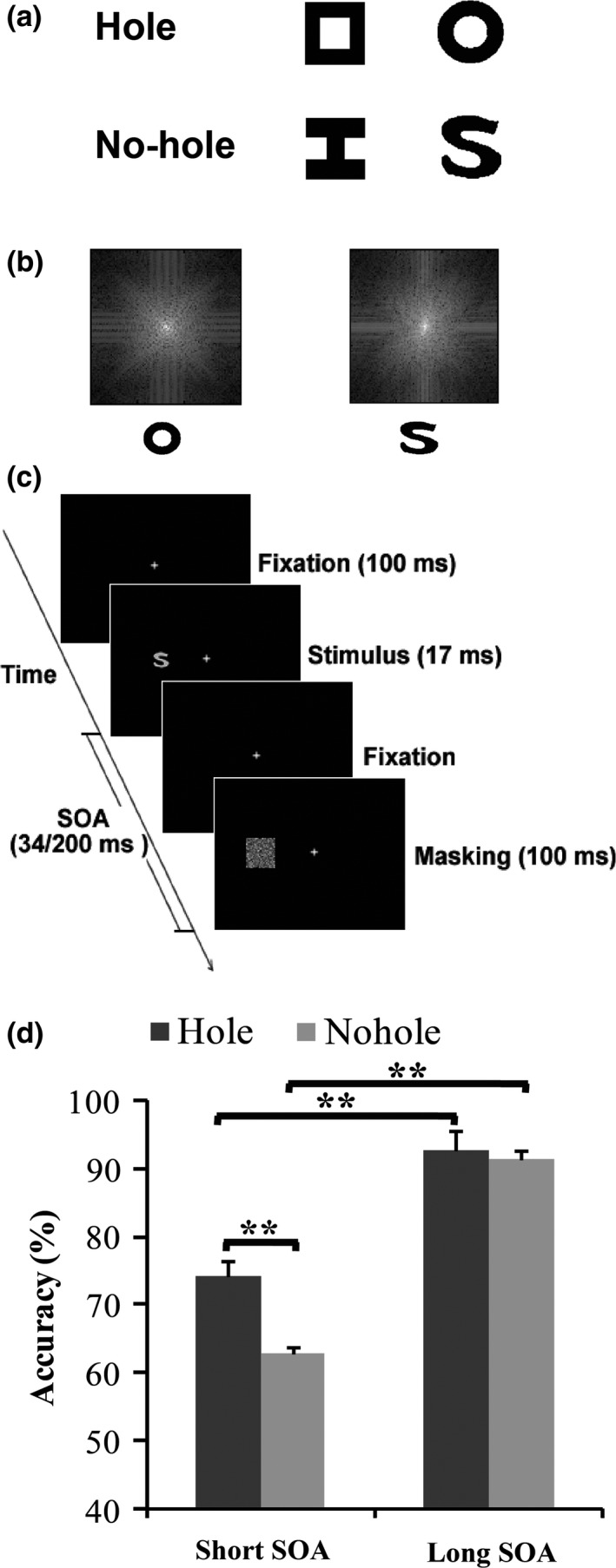
(a) Schematic depiction of the stimulus pairs used as target stimuli. (b) The power spectra (2D Fourier transformation) of the ring versus the S. (c) Schematic illustration of the visual task in the fMRI experiments. (d) Mean discrimination performance of the hole stimulus and no‐hole stimulus in long and short SOA conditions. Error bars indicate the standard error of the mean (*SEM*). **p < .01. fMRI, functional magnetic resonance imaging; SOA, stimulus onset asynchrony

As shown in Figure 1a, to rule out the confounds from local features, we carefully designed the stimuli to minimize the difference in low‐level physical features between the “hole” and “no‐hole” stimuli. The target images consisted of two hole stimuli (a 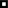
‐shaped figure and a ring) and two no‐hole stimuli (a 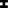
‐shaped figure and an S‐shaped figure). The 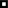
‐shaped and 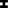
‐shaped figures (hereafter referred to as “
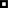
” and “
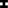
”) were designed to have equal luminous flux and equal average edge crossings. The ring and the S‐shaped figure (hereafter referred to as O and S), were designed not only to have equal luminous flux and equal average edge crossings, but also to have nearly equal perimeter lengths and equal spatial frequency components (Figure 1b) (Chen et al., 2003).

As illustrated in Figure 1c, each trial began with a background containing the fixation points subtending 0.2º × 0.2º. Participants were instructed to focus their gaze on the fixation point. All stimuli were drawn gray on a black background. The target subtended (1.8º × 1.8º) and was presented to the left or right of fixation. The mask was a Gaussian noise square (3.3º × 3.3º) that covered the area of the target. The distance between centers of the stimuli and the fixation was 3.5º. As shown in Figure 1c, in each trial (except the mask‐only trial and null trial), after a 400‐ms fixation interval, a target was presented for 17 ms, and immediately followed by a mask presented for 100 ms for one of two possible SOAs: 34 ms (short SOA condition), or 200 ms (long SOA condition). After the mask disappeared, the fixation was presented again and remained visible until the start of the next trial. In the mask‐only trial, only a mask was presented without a preceding target (mask‐only condition), which was designed to exclude possible response bias. In the null trial, only a fixation was presented throughout the trial (baseline condition). Participants were asked to press the button as accurately as possible whenever a target stimulus was discriminate and to withhold responses on catch trials with no target. The stimuli pair and SOA were randomized and counterbalanced across runs.

The stimuli were presented with MATLAB using the Psychophysics Toolbox through a LCD projector onto a rear projection screen, which was located behind the participant's head inside the magnet bore and were viewed through a mirror on the head coil.

### 2.3 Design

The fMRI study used the rapid event‐related design, in which MRI images from eight scans were collected. Each run lasted 408 s and had two 12‐s fixation periods, one at the beginning and one at the end. Between the two fixation periods, a total of 128 trials were presented to participants at a rate of one every 3 s, 32 each of the four conditions: two stimuli conditions (“hole” trials and “no‐hole” trials), mask‐only trials, and the null trials. For each run, trial order was randomized and counterbalanced using M‐sequences (Buracas, Fine, & Boynton, 2005).

### 2.4 Data acquisition

Data were acquired with a 3T scanner (TRIO; Siemens) using a 12‐channel head coil. For all participants, the functional images were collected using a gradient‐echo echo planar imaging (EPI) sequence (25 contiguous axial slices; slice thickness = 4 mm; interslice gap = 1 mm; FOV = 220 × 220 mm; acquisition matrix = 64 × 64; voxel size = 3.4 × 3.4 × 4 mm; TR = 1,500 ms; TE = 29 ms; flip angle = 90), and the high‐resolution T1‐weighted structural images were collected using an MPRAGE (magnetization prepared rapid gradient echo) sequence (1 × 1.3 × 1 mm resolution).

### 2.5 Data analysis

All preprocessing and most statistical analyses were performed using the software package SPM2 (Welcome Department of Cognitive Neurology, London) and MarsBaR toolbox (Brett, Anton, Valabregue, & Poline, 2002). The EPI images were temporally corrected to the middle slice, then were spatially realigned, and normalized to a standard Montreal Neurological Institute (MNI) reference brain in Talairach space, and finally were smoothed with an isotropic 8‐mm Gaussian kernel.

First, the early visual cortex (Brodmann's 17 and 18 areas) ROI (473 average voxels) was identified by the group results of neural activation for mask‐only condition over baseline condition, masked by the template defined by automated anatomical labeling map (AAL) (Tzourio‐Mazoyer et al., 2002). The priori ROIs, LGN, SC and pulvinar anatomical ROIs were created based on pertinent literature (Kastner et al., 2002; Morris, de Gelder, Weiskrantz, & Dolan, 2001) and defined as a sphere of 5 mm radius (133 voxels) respectively (Hsu, et al. 2013; Schmid et al., 2013; Steuwe, et al., 2015). The anatomical ROIs of the left and right LGN were centered at (*x* = 23, *y* = −21, *z* = −5, *x* = −23, *y* = −21, *z* = −5). The SC anatomical ROI was centered at (*x* = 0, *y* = −36, *z* = −8). The left and right pulvinar anatomical ROIs were centered at (*x* = 17, *y* = −24, *z* = 12; *x* = −12, *y* = −24, *z* = 8).

Time courses of each condition were extracted from each ROI using the MarsBar toolbox to compute their respective percentage signal changes, first within each subject and subsequently averaged across subjects. The resulting analysis produced a 9‐lag (13.5 s) time course for each condition. The percentage signal change of each time course was normalized to the first image acquired after stimulus presentation (baseline) (Todd & Marois, 2004). Then, the percent of signal change was measured at the peak of time course (Gregorios‐Pippas, Tobler, & Schultz, 2009) (6 s after stimulus in early visual cortex, LGN and pulvinar, 4.5 s after stimulus in SC (DeSimone, Viviano, & Schneider, 2015)).

## 3 RESULTS

### 3.1 Behavioral results

The mean false‐alarm rates on catch trials were 3%. A two‐way repeated‐measures ANOVA was conducted for SOA conditions (34 and 200 ms) and target types (hole or no‐hole stimuli) as within‐subject factors. The mean accuracies were analyzed using a paired *t* test with Bonferroni correction, respectively (Bland & Altman, 1995). A value of .05 was chosen as the significant level, and it was divided by the number of pairwise comparisons (4 [Short SOA condition: hole vs. no‐hole; Long SOA condition: hole vs. no‐hole; Hole: short SOA vs. long SOA; no‐hole: short SOA vs. long SOA]). Thus, a significant level of .125 was used.

As shown in Figure 1d, the results showed a significant main effect of the SOA condition, *F*(1, 18) = 96.85, *p* < .001, and a significant main effect of the target type, *F*(1, 18) = 17.22, *p* = .001. Importantly, there was a significant interaction between SOA condition (short and long SOA) and target type (“hole” and “no‐hole”), *F*(1, 18) = 9.33, *p* = .007, suggesting that the backward masking had a different impact on the hole and the no‐hole perceptions. Under the long SOA condition, the hole and no‐hole stimuli were both well discriminated (93% and 92% for the hole and no‐hole stimuli, respectively), and no difference was found between them, *t*(18) = 0.96, *p* = .35. Compared with the long SOA condition, participants' performances were significantly impaired under the short SOA condition for both hole and no‐hole stimuli, hole: *t*(18) = −6.24, *p* < .001; no‐hole: *t*(18) = −9.93, *p* < .001. However, further analysis revealed that subjects showed better perception of hole stimuli than no‐hole stimuli under strong masking conditions, *t*(18) = 3.82, *p* = .001.

### 3.2 ROI results

**Figure 2:**
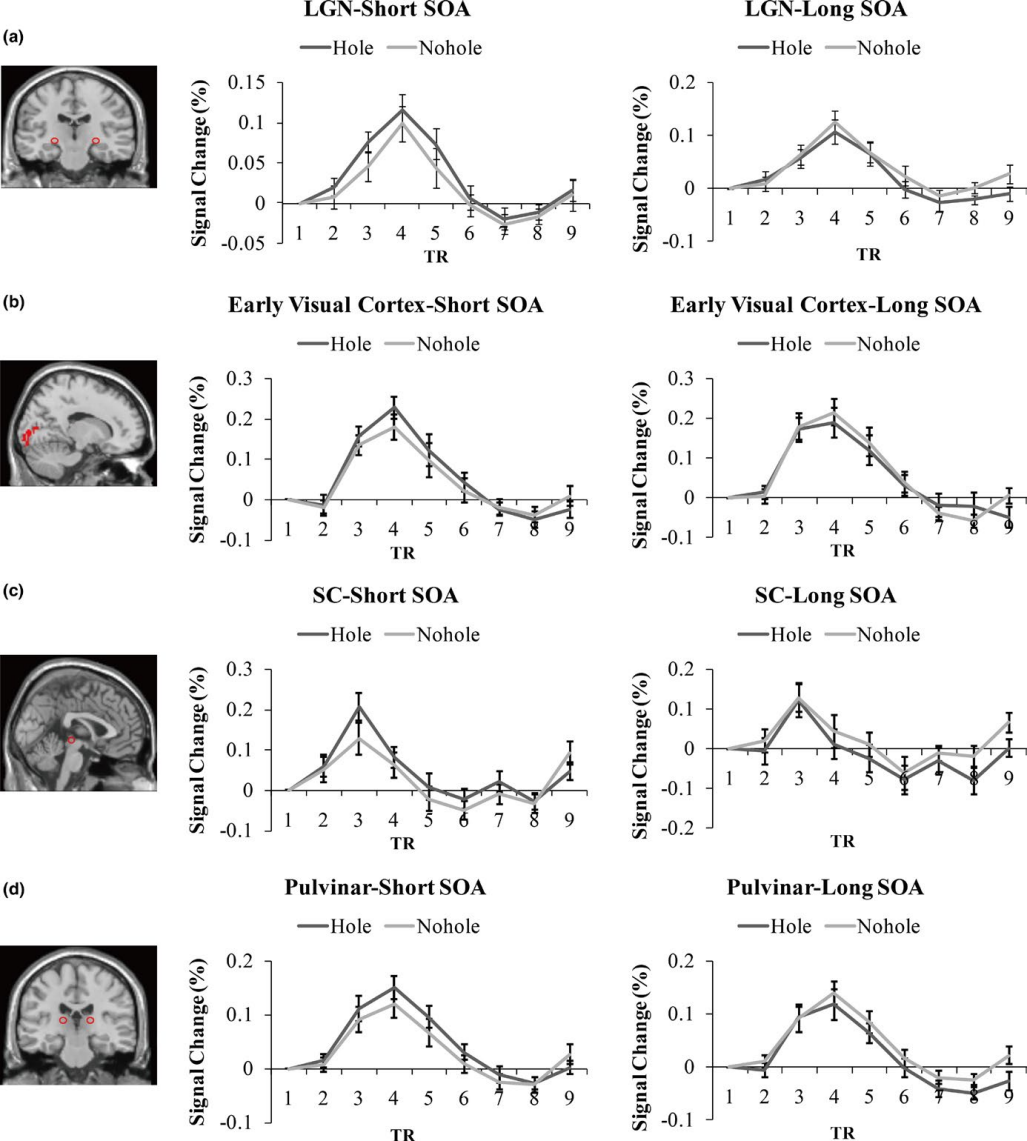
Time courses of the hemodynamic response in regions of interest. Time courses of the hemodynamic response for each stimulus condition (In left panel, Short SOA condition: hole vs. no‐hole; In right panel, Long SOA condition: hole vs. no‐hole) for the event‐related runs averaged across participants are shown for the LGN (a), early visual cortex (b), SC (c) and pulvinar (d). Error bars represent *SEM*. LGN, lateral geniculate nucleus; SC, superior colliculus; SOA, stimulus onset asynchrony

As shown in Figure 2, the average time course of percent signal change in four ROIs (LGN, early visual cortex, SC, and pulvinar) was extracted from the event‐related scans of each subject for all the trial conditions (Short SOA condition: hole vs. no‐hole; Long SOA condition: hole vs. no‐hole).

Consistent with previous studies, the peak response in LGN, early visual cortex, and pulvinar occurred at a latency of 6 s, while in SC at 4.5 s (DeSimone et al., 2015) after the beginning of the trial. The magnitude of this peak response was analyzed in a three‐way repeated‐measures ANOVA across subjects with target type (hole, no‐hole), SOA condition (short SOA: 34 ms; long SOA: 200 ms), and ROI site (LGN, early visual cortex, SC, and pulvinar) as repeated‐measures variables.

The main effects of ROI site and target type were both significant, ROI site: *F*(3,54) = 5.96, *p* = .001; target type: *F*(1,18) = 5.67, *p* = .03. The main effect of the SOA condition was not significant, SOA condition: *F*(1,18) = 2.81, *p* = .11. There was a borderline significant two‐way interaction between ROI site and target type, *F*(3,54) = 2.56, *p* = .07. The interactions between ROI site and SOA condition, *F*(3,54) = 3.44, *p* = .03, and between target type and SOA condition, *F*(1,18) = 16.73, *p* = .001, were both significant. Importantly, a significant three‐way interaction of target type × SOA condition × ROI site was obtained, *F*(3,54) = 2.92, *p* = .04. To unravel this interaction, separate analyses were performed for each ROI. For each ROI, the data were entered into a two‐way repeated‐measures ANOVA with SOA condition (short SOA: 34 ms; long SOA: 200 ms) and target type (hole, no‐hole) as within‐subject factors. The post hoc multiple comparisons were corrected using the Benjamini–Hochberg (BH) procedure with *q* values (FDR) ≤0.05 as cutoffs (Benjamini & Hochberg, 1995). Corrected *p*‐values were used.

**Figure 3:**
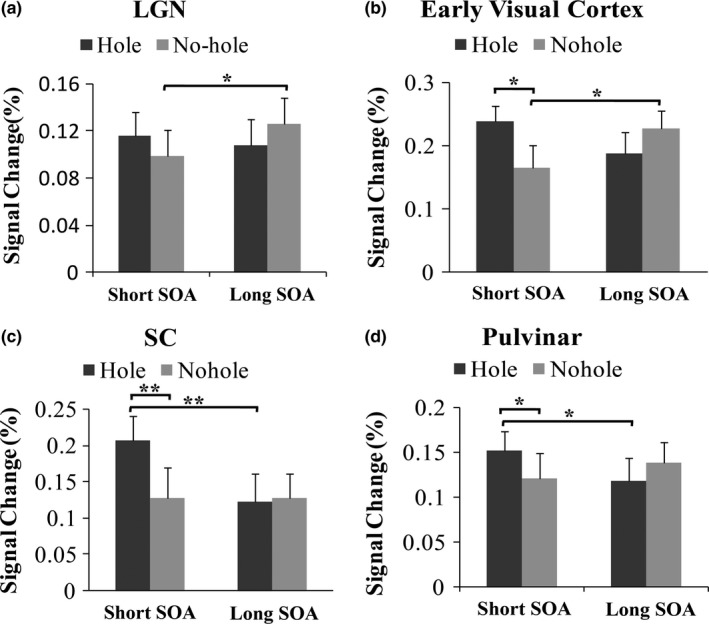
The mean peak bold oxygen level‐dependent (BOLD) signal intensity in LGN (a), early visual cortex (b), SC (c) and pulvinar (d). Error bars represent *SEM*. *p < .05, **p < .01. LGN, lateral geniculate nucleus; SC, superior colliculus

As shown in Figure 3a, the main effects of SOA condition and target type in LGN were not significant, SOA condition: *F*(1,18) = 1.33, *p* = .26; target type: *F*(1,18) = 0.01, *p* = .93. However, a significant interaction was found between SOA condition and target type, *F*(1,18) = 6.4, *p* = .02. The prior paired sample post hoc analysis found that whereas the neural response to no‐hole stimuli was significantly decreased under the short SOA condition, *t*(18) = −2.64, *p* = .038, when it was rendered more invisible by strong masking, no difference was found in the LGN for hole stimuli between two different SOA conditions, *t*(18) = 0.73, *p* = .54. In two SOA conditions, no significant difference was found between the neural response of hole and no‐hole stimuli, Short SOA: hole vs. no‐hole, *t*(18) = 1.45, *p* = .2; Long SOA: hole vs. no‐hole, *t*(18) = −1.53, *p* = .19.

In the early visual cortex (Figure 3b), the main effects of SOA condition and target type were not significant, SOA condition: *F*(1,18) = 0.14, *p* = .71; target type: *F*(1,18) = 1.53, *p* = .23. Importantly, a significant interaction was observed between SOA condition and target type, *F*(1,18) = 12.02, *p* = .003. Further post hoc analysis revealed that whereas the neural activity of the early visual cortex was greatly reduced, *t*(18) = −3.09, *p* = .02 for no‐hole stimuli, when no‐hole stimuli was significantly suppressed by strong masking. No difference was observed between the two SOA conditions for hole stimuli, *t*(18) = 1.82, *p* = .09, *q* = 0.14. Moreover, under the short SOA condition, the neural response to hole stimuli was significantly stronger than to no‐hole stimuli in early visual cortex, Short SOA condition: hole vs. no‐hole, *t*(18) = 3.43, *p* = .016. Under the long SOA condition, no difference was found between hole and no‐hole stimuli, Long SOA condition: hole vs. no‐hole, *t*(18) = −1.83, *p* = .15.

In SC, the main effects of SOA condition and target type were both significant, SOA condition: *F*(1,18) = 9.37, *p* = .007; target type: *F*(1,18) = 10.16, *p* = .005, (Figure 3c). Importantly, there was a significant interaction between SOA condition and target type, *F*(1,18) = 7.83, *p* = .01. A prior post hoc test was performed, and we found that the neural activity for hole stimuli significantly increased and stimulus perception greatly decreased by strong masking, *t*(18) = 4.39, *p* = .006, for the short SOA condition. For no‐hole stimuli, no difference was found between the two SOA conditions in SC, *t*(18) = 0.046, *p* = .96. Similar to the ROI results of the early visual cortex, the neural response in SC to the hole stimuli was greater than the response to no‐hole stimuli, Short SOA condition: hole vs. no‐hole, *t*(18) = 4.17, *p* = .005, under strong masking. No difference was found between the two target stimuli under the long SOA condition, Long SOA condition: hole vs. no‐hole, *t*(18) = −0.27, *p* = .84.

Figure 3d shows that the main effects of SOA condition and target type in pulvinar were not significant, SOA condition: *F*(1,18) = 1.19, *p* = .28; target type: *F*(1,18) = 0.46, *p* = .51. Importantly, a significant interaction was observed between SOA condition (34 and 200 ms) and target type (hole and no‐hole), *F*(1,18) = 10.39, *p* = .005. Further analysis revealed that under the short SOA condition, the neural activity to hole stimuli increased significantly, *t*(18) = 3.27, *p* = .017, when the stimulus visibility was greatly attenuated because of masking. For no‐hole stimuli, no difference was observed in pulvinar between the two SOA conditions, *t*(18) = −1.67, *p* = .16. A significantly stronger neural response to hole stimuli was obtained in pulvinar compared with no‐hole stimuli, Short SOA condition: hole vs. no‐hole, *t*(18) = 2.88, *p* = .027. There was no detectable difference between these two stimuli under the long SOA condition, Long SOA condition: hole vs. no‐hole, *t*(18) = −1.9, *p* = .15.

In summary, for no‐hole stimuli, the neural activation of the LGN and the early visual cortex was greatly attenuated when the no‐hole perception was impaired by strong masking. For hole stimuli, no suppressed neural activation in the LGN and the early visual cortex was observed under poor visibility induced by masking. On the contrary, an enhanced neural response to hole stimuli in the SC and the pulvinar was obtained when the stimulus was rendered more invisible. Moreover, the neural responses to hole stimuli in the early visual cortex, the SC, and the pulvinar were significantly stronger than the responses to no‐hole stimuli.

## 4 DISCUSSION

The present study investigated the neural process for the salience of hole and no‐hole features using a backward masking paradigm. Whereas both hole and no‐hole stimuli were readily detected with a long SOA, the visibilities of hole and no‐hole stimuli were both attenuated with a short SOA, revealing a strong suppression effect on target perception. Similarly, the fMRI results revealed a decreased neural activity in response to no‐hole stimuli in the LGN and early visual cortex under the short SOA condition. The ROI result is consistent with previous findings, which have shown that backward masking can suppress conscious perception of targets and attenuate neural activation of the visual pathway, possibly as early as in the LGN and the early visual cortex (Lamme et al., 2002). The behavioral and ROI results indicated that the visual awareness of no‐hole stimuli was correlated with the brain activity of the cortical route via the LGN to the early visual cortex.

Although the perception of hole and no‐hole was both impaired by strong masking, hole features were significantly better discriminated than no‐hole stimuli. The behavioral results revealed a priority for hole perception under the low awareness condition. Further ROI analysis indicated that strong masking did not suppress the neural response to hole stimuli in the LGN and early visual cortex. Instead, strong masking induced enhanced neural activity in the SC and pulvinar. Moreover, the ROI analysis revealed that the neural signal of hole stimuli in the early visual cortex, the SC, and the pulvinar was significantly greater than no‐hole stimuli under strong masking.

Based on the behavioral and ROI results, we formulated a hypothesis for the mechanism yielding a priority for the global topological perception of holes. As stated in previous studies, the global topological property is the primitive of visual perception. The hole is a global topological feature and has priority during processing and recognition by the human visual system even under poor visibility conditions, as reported in the present study. These results suggest that when the perceptual visibility of holes is greatly suppressed, a subcortical route might be involved in enhancing the activation of the SC and pulvinar, thus strengthening the information of holes to overcome the suppression induced by masking. Previous studies have shown that there is a connection between the pulvinar and the cortical visual area (Tamietto & de Gelder, 2010). Therefore, it is possible that the early visual cortex, which also receives the strengthened hole information from the pulvinar, induces a stronger neural activation to hole stimuli than to no‐hole stimuli under the short SOA condition. Enhanced activation of the subcortical pathway enables quicker processing of hole features. However, the suppression induced by the backward masking could happen at multiple levels in the brain. The hole stimuli could be processed via a fast subcortical route. Yet, it could not completely escape from the higher cortical suppression, which resulted in impaired hole perception. However, further investigations are needed to clarify this issue.

One might argue that the difference of the stimulus in the low‐level features (i.e., orientation and spatial frequency) could also contribute to the difference in neural activity. This alternative explanation could be ruled out, however, by controlling for the low‐level feature differences between the hole and no‐hole stimuli as much as possible. Indeed, there can be no two geometric figures that differ only in topological properties (i.e., the presence or absence of a hole), without any differences in nontopological factors. Thus, we could not test for the role of the hole feature in the absence of awareness in complete isolation. We minimized this problem through systematical and careful design of the stimulus pair to prevent subjects from using nontopological properties, including spatial frequency components and the number of edges crossed while scanning a figure, to perform the task. For instance, “
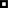
” and “
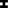
” were designed to have equal areas (and therefore luminous flux) and equal average edge crossings. The O and S were made to have equal luminous flux, nearly identical spatial frequency components and perimeter length, and equal average edge crossings. The current findings cannot be consistently explained by low‐level feature differences under such converging operations. The topological account is the only one that explains, in a unified manner across all stimulus pairs used, a privileged detection of “hole”.

It is well established that whereas the classical cortical visual pathway mainly processes slow and detailed visual information for subsequent conscious perception, the subcortical pathway is mainly responsible for processing coarse, unawareness, and early visual information (Pasley et al., 2004). The present study provides direct neural evidence demonstrating that these two distinct visual pathways mediate hole and no‐hole processing, respectively. For no‐hole stimuli, a cortical route, via the LGN and early visual cortex, is required to “drive” the perceptual response. For hole stimuli, the subcortical route through the SC and pulvinar is involved, especially under low visual awareness levels.

Combined with previous studies, the current findings suggest that perceiving the global topological property of holes might be a fundamental function of the visual system, which require the involvement of the subcortical route and thereby results in rapid and preferential recognition. Furthermore, the rapid subcortical processing of hole features might provide a new direction for the understanding of the neural correlates of the “global‐first” topological approach in primitive visual perception.

## CONFLICT OF INTEREST

None declared.
